# Identifying at risk populations amongst breast cancer survivors and their common symptoms and concerns

**DOI:** 10.1186/s12905-023-02458-1

**Published:** 2023-06-08

**Authors:** Pooja Roy, Iuliia Kovalenko, Janet Chan Gomez, Kit Lu, Beth Rudge, Yijin Wert, Lisa Torp

**Affiliations:** 1 University of Pittsburgh Medical Center (UPMC) Harrisburg Hospital, 205 S Front St, Suite 3C, Harrisburg, PA 17104 USA; 2 UPMC Hillman Cancer Center, 4300 Londonderry Rd, Suite 201, Harrisburg, PA 17109 USA

**Keywords:** Breast cancer survivorship, Return to baseline, Quality of life, Vulnerable population, Common concerns

## Abstract

**Purpose:**

The survival rate amongst breast cancer survivors (BCS) have been increasing, with a 5-year survival rate of almost 90%. These women face many quality of life (QOL) issues either due to either cancer itself or the complex treatment regimen. Our retrospective analysis aims to identify at risk populations among the BCS and their most common concerns.

**Methods:**

This is a single-institution, retrospective, descriptive analysis of patients who were seen at our Breast Cancer Survivorship Program from October 2016 to May 2021. Patients completed a comprehensive survey which assessed self-reported symptoms, their concerns and degree of worry and recovery to baseline. The descriptive analysis on the patient characteristics included age, cancer stage and treatment type. The bivariate analysis included the relationship between the patient characteristics and their outcomes. Analysis of group differences was completed with Chi-square test. When the expected frequencies were five or less, Fisher exact test was used. Logistic regression models were developed to identify significant predictors for outcomes.

**Results:**

902 patients (age 26–94; median 64) were evaluated. Majority of women had stage 1 breast cancer. The most common self-reported concerns affecting the patients were fatigue (34%), insomnia (33%), hot flashes (26%), night sweats (23%), pain (22%), trouble concentrating (19%), and neuropathy (21%). Though 13% of BCS felt isolated at least 50% of their time, the majority of patients (91%) reported having a positive outlook and felt that they have a sense of purpose (89%). Younger patients were more likely to worry about their cancer more than 50% of the time (p < 0.0001). Patients that were less likely to return back to at least 50% of their pre-treatment baseline were younger (age ≤ 45) (p = 0.0280), had higher stage breast cancer (Stage 2–4) (p = 0.0061), and had chemotherapy either alone or as part of their multi-modality treatment (p < 0.0001).

**Conclusion:**

According to our study, younger patients, those with higher stage breast cancer and survivors who had chemotherapy may experience significant QOL issues. Fortunately, majority of BCS report a positive and optimistic outlook post treatment. Identifying common concerns after treatments and vulnerable populations are especially important to deliver quality care and to optimize interventions.

**Implications for Cancer Survivors:**

Our study identified the most common self-reported concerns affecting BCS. In addition, our results suggest that younger patients, patients with higher stage breast cancer and survivors who had chemotherapy were more likely to have QOL issues. Despite this, our study showed, the majority of BCS reported positive outlooks and emotions.

## Introduction

Breast cancer is the most common malignancy in women affecting one out of eight women. Fortunately, the death rate among female breast cancer patients has dramatically decreased due to the advances in treatment [1, 2]. This has led to an average 90% five-year survival rate among female patients with breast cancer [2], or approximately four in ten female cancer survivors in the United States [2]. Due to the increasing prevalence of breast cancer survivors (BCS), an area of research has emerged which addresses health, wellness and quality of life (QOL) in post-treatment cancer survivors. Increased attention into post-cancer care is vital due to the rapidly growing population of survivors and their unmet needs.

The impact of breast cancer treatment not only affects physical well-being, but often psychological, social and spiritual health are impacted as well. Physical consequences include fatigue, sleep, infertility, decline in functional status and pain [3]. Mental health issues including anxiety, depression and fear of recurrence also plague survivors [3]. These effects extend into their social lives, causing family and relationship distress, sexual dysfunction, isolation and financial burden [3]. Breast cancer survivors in particular experience upper arm numbness, bone loss, hot flashes, body image concerns, change in sexual desire and chronic pain [3]. The impact of certain symptoms can be related to various patient factors such as age, breast cancer diagnosis, stage at the time of diagnosis, treatment, family support and other medical comorbidities. Although breast cancer is more prevalent in patients older than 65 years of age, younger breast cancer survivors may face a unique set of challenges after treatment of their cancer. Therefore, our retrospective analysis aims to identify at risk populations in breast cancer survivors, and their common side effects and quality of life issues.

## Methods

### Study design, cohort and setting

This is a single-institution, retrospective, descriptive analysis of patients who were seen at our Breast Cancer Survivorship Program from October 2016 to May 2021. Survivors were seen at our Survivorship Program approximately six months after their initial treatment (such as surgery or completion of chemotherapy or radiotherapy) as part of our clinical initiatives to address patients’ concerns early during their survivorship journey.

### Program description

Our program enrolls breast cancer survivors six months after initial treatment. Patients were asked to fill out comprehensive surveys when meeting with nurse practitioners during enrollment. The purpose of the questionnaire was to facilitate discussion of patients’ symptoms and concerns during their survivorship clinical visit. It served as a starting point for a more in-depth discussion and assessment with the Survivorship provider. After a thorough clinical assessment, the survivorship clinical provider would then prescribe the necessary treatment recommendations or specialty referrals. Examples of treatment recommendations include both pharmacologic and nonpharmacological modalities. Our program offers multimodal and integrative treatment such as yoga, acupuncture, and exercise. Depending on patients’ concerns, specialty referrals including lymphedema, physical therapy, counseling/therapy, sleep medicine, or other medical specialties. In addition, survivorship follow-up was offered as needed or their concerns were directed back to the patients’ primary treatment team.

### Study outcome measure

Patients were given a comprehensive survey to assess self-reported symptoms and concerns following their treatment. Our survey evaluated the most common symptoms related to cancer treatment (fatigue, insomnia, hot flashes, night sweats, pain, trouble concentrating, and neuropathy). Each symptom was scored 1 to 5, for the amount of distress or concern it afflicted. Symptoms with a score of 3 or more were considered significant. Patients also reported how often they spent thinking or worrying about their cancer (0%, 25%, 50%, 75%, 100% of the time). Data was then grouped into less than 50%, and more than or equal to 50% of the time. Feeling back to pre-treatment baseline served as the main subjective to assess patients’ perception of their quality of life with emotional aspects being the supplemental subjective measures (feeling happy, purpose, satisfied, in control, useful, worried, sad, isolated, and hopeless). These subjective measures were included if they affected the patient at least half of their time.

### Statistical analysis

The descriptive analysis on the patient characteristics included age, cancer stage and treatment type. The bivariate analysis included the relationship between the patient characteristics and their outcomes. Chi-square test was used to analyze group differences. The Fisher exact test was employed when any of the expected frequencies was five or less. Logistic regression models were developed to identify significant predictors for outcomes. Odds ratios and their 95% confidence interval were reported for each predictor. A p-value of less than 0.05 was considered statistically significant. All the analyses were done by SAS version 9.4 (SAS institute, Cary NC).

## Results

### Patient characteristics

Our study included 902 patients. Their ages ranged from 26 to 94, with a median age of 64.

### Patient cancer characteristics and treatment

Patients were diagnosed with breast cancer stage 0 to 4. They were diagnosed with cancer between the years 2017 and 2020. The majority of patients had stage 1 (59.5%), followed by stage 0 (19.0%) and stage 2 breast cancer (15%). The least represented were among those with stage 3 (6.3%) and stage 4 (0.2%) breast cancer. The survivors had undergone varying treatments, with the most common being endocrine therapy and radiation (27.3%). This was followed by endocrine therapy, radiation and chemotherapy combined (18.2%). A portion of patients had endocrine therapy only (16.7%) or surgery only (12.4%). The rest of the patients had radiation alone (7.1%), radiation with chemotherapy (7.0%), endocrine with chemotherapy (6.9%) or chemotherapy alone (4.4%).

### Participant symptoms

**Fig. 1:**
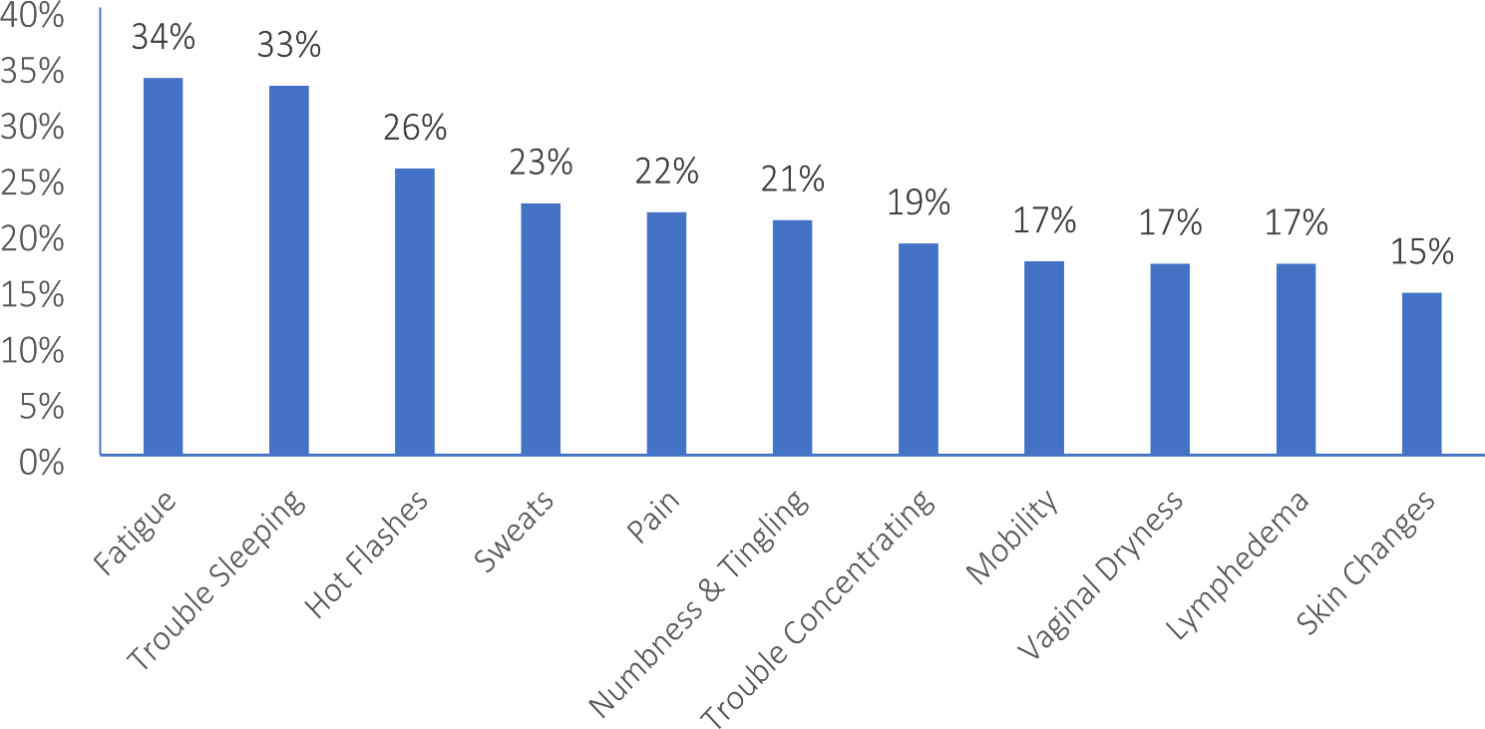
Self-reported concerns affecting BCS

**Fig. 2:**
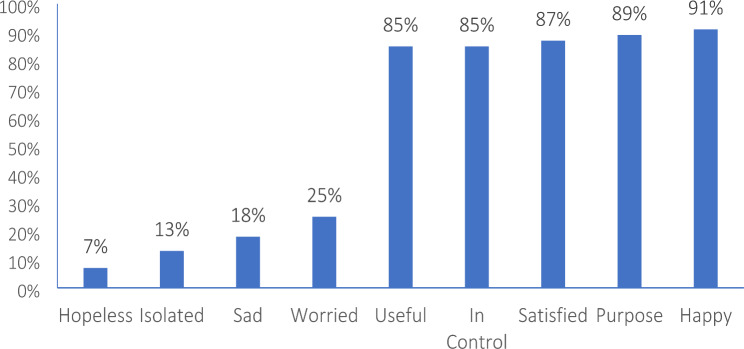
Self-reported feelings affecting BCS more than half of their time

Most common self-reported concerns affecting BCS were fatigue (34%), insomnia (33%), hot flashes (26%), night sweats (23%), pain (22%), neuropathy (21%) and trouble concentrating (19%) [Fig. 1]. Less commonly reported symptoms were mobility issues (17%), vaginal dryness (17%) and skin changes (15%) [Fig. 1]. The majority of patients (91%) reported having a positive outlook and felt that they have a sense of purpose (89%). They reported feeling satisfied (87%), in control (85%) and useful (85%). However, a quarter of patients felt worried. Other self-reported feelings of sadness, isolation and hopelessness were less reported (18%, 13% and 7% respectively) [Fig. 2]. Patients who worried more than 50% of the time (p < 0.0001) and were feeling 50% or less back to baseline (< 0.0001) had higher number of symptoms on average [Table 1].

**Table 1:** Symptom burden in patients worrying about cancer and not returning to 50% of their baseline

	Number of significant symptoms						p-Value
	Total number of patients	Mean	Std Dev	Minimum	Maximum	Median	
Patients worrying about cancer > 50% of the time	142	4.20	3.04	0	11	4	< 0.0001
Patients worrying about cancer </= 50% of the time	721	2.07	2.37	0	11	1	
Patients feeling </= 50% back to treatment baseline	209	4.19	2.93	0	11	4	< 0.0001
Patients feeling > 50% back to treatment baseline	603	1.82	2.21	0	10	1	

### Worrying about cancer

The group with higher cancer stages (stage 2–4) (p = 0.0007), age 45 and younger (< 0.0001) and who had chemotherapy as monotherapy or in combination (p = 0.0157) had higher percentages of patients who worried about cancer more than 50% of the time [Table 2]. After multiple logistic regression analysis only younger patients (p < 0.0001) and those who did not return to more than 50% of their baseline (p < 0.0001) were significantly more likely to worry about cancer [Table 3].

**Table 2:** Patient worrying about cancer 50% or more of the time by cancer stage, by age group and by treatment

	Total number of Patients	Number of patients worrying about cancer > 50%	Percent of patients worrying about cancer > 50%		p-Value	
Cancer Stage					0.0007	
0	161	26	16.15%			
1	513	68	13.26%			
2	132	37	28.03%			
3 or 4	56	11	19.64%			
Age Group					< 0.0001	
Age 45 and younger	78	38	48.72%			
Age 46–69	519	84	16.18%			
Age 70 and older	266	20	7.52%			
Treatment					0.0157	
Endocrine only	146	23	15.75%			
Radiation only	57	4	7.02%			
Chemotherapy only	40	7	17.50%			
Endocrine and Radiation	236	27	11.44%			
Endocrine and Chemo	61	16	26.23%			
Radiation and Chemo	61	13	21.31%			
Endocrine, Radiation and Chemo	157	35	22.29%			
None of the three treatments	105	17	16.19%			

**Table 3:** Multiple Logistic Regression: Predictors for patient worrying about cancer 50% of the time or more

Predictors	Odds Ratio	95% Confidence Interval		p-Value
		Lower Limit	Upper Limit	
Age 45 and younger	4.99	2.90	8.59	< 0.0001
Cancer Stage 2–4	1.42	0.86	2.32	0.1707
Chemo alone or as a part of their multi-modality treatment	1.07	0.67	1.72	0.7688
Returning back to baseline 50% or less	3.86	2.53	5.90	< 0.0001

### Returning back to 50% of baseline

The group with higher cancer stages (stage 2–4) (p < 0.0001), age 45 and younger (p = 0.0001) and who had chemotherapy as monotherapy or in combination (p < 0.0001) had higher percentages of patients who felt less than 50% of their baseline [Table 4]. Multiple logistic regression analysis confirmed that young cancer survivors (age ≤ 45) (p = 0.028), higher stage BCS (Stage 2–4) (p = 0.0061), and those who had chemotherapy either alone or as part of their multi-modality treatment (p < 0.0001) were significantly less likely to return back to at least 50% of their pre-treatment baseline [Table 5].

**Table 4:** Patient feeling 50% or less back to pre-treatment baseline by cancer stage, by age group and by treatment

	Total number of patients	Number of patients feeling 50% or less back to treatment baseline	Percent of patients feeling 50% or less back to treatment baseline	p-Value		
Cancer Stage				< 0.0001		
0	153	25	16.34%			
1	477	108	22.64%			
2	127	49	38.58%			
3 or 4	54	27	50.00%			
Age Group				0.0001		
Age 45 and younger	75	32	42.67%			
Age 46–69	492	131	26.63%			
Age 70 and older	245	46	18.78%			
Treatment				< 0.0001		
Endocrine only	129	21	16.28%			
Radiation only	58	15	25.86%			
Chemotherapy only	36	14	38.89%			
Endocrine and Radiation	225	35	15.56%			
Endocrine and Chemo	59	26	44.07%			
Radiation and Chemo	59	23	38.98%			
Endocrine, Radiation and Chemo	147	54	36.73%			
None of the three treatments	99	21	21.21%			

**Table 5:** Multiple Logistic Regression: Predictors for patients returning back to baseline 50% or less

Predictors	Odds Ratio	Lower Limit	Upper Limit	p-Value
Age 45 and younger	1.78	1.06	2.97	0.0280
Cancer Stage 2–4	1.74	1.17	2.59	0.0061
Chemo alone or as a part of their multi-modality treatment	2.23	1.55	3.20	< 0.0001

## Discussion

Our study demonstrated that young cancer survivors (age < 45) are not only less likely to return to pre-treatment baseline (p = 0.028) but are also more likely to worry about their cancer (p < 0.0001). This at-risk age group was important to identify as clinicians may identify older patients to be more vulnerable [2, 3]. Recent publications have been focused on the patients’ age as a major predisposing factor in determining vulnerable patient population [4, 5]. Previous studies demonstrate controversial results with some data supporting our findings [5] and others emphasizing vulnerability of the older population [4]. Concerns amongst young BCS may be attributed to a variety their age-related needs such as fertility, sexual performance, sensitivity to social deprivation and need to return to work [6]. Wherein, career and socio-economic status are reported to be among the most concerning issues causing distress in young patient population [7–9]. Quality of life of young BCS can also be dramatically affected by their sensitivity to decreased physical performance secondary to treatment-related health issues. Although older population is more vulnerable to side effects of anticancer treatment, younger patients usually experience more significant decrease from their baseline functional status [8]. Fatigue, insomnia, and hot flashes may raise no concerns in older BCS as these symptoms can be attributed to normal aging. In addition, younger patients have more difficulty adjusting psychologically as they are less likely to expect a diagnosis of cancer, acknowledge the aggressiveness of the disease, intensity of the treatment plan and are more likely to be preoccupied by their potential life expectancy. Similarly, fertility-related concerns are reported as one of the most distressing factors related to treatment adverse effects [6]. This younger patient population are usually offered multi-modal treatment approaches, which lead to swift onset of menopause and affect reproductive health throughout the patient’s life [10]. In light of this data, greater attention should be directed to the younger BCS population during post-treatment follow ups. Addressing these issues prior to treatment may also help prepare younger patients to these challenges.

The other two at risk groups identified by our study included patients with higher stage breast cancer (stage 2–3) and those who underwent chemotherapy. Patients with higher stage breast cancer (p = 0.0061) and who underwent chemotherapy alone or as part of their multi-modal treatment (p < 0.0001) were less likely to return to baseline. These findings are expected as patients with higher stage breast cancer commonly require more aggressive treatment, including chemotherapy that have several detrimental effects physically and emotionally. These findings have been supported by various studies including a systematic review demonstrating distress being significantly associated with advanced cancer at diagnosis and treatment with chemotherapy [5]. In addition, a comparative cross-sectional study showed an inverse correlation between QoL and stage of breast cancer and chronic disease in the chemotherapy group [11]. Whereas other studies, such as the article published in the Annals of Oncology concluded that endocrine therapy, but not chemotherapy was detrimental on QoL as measured by C30-SumSc [12]. Regardless, identifying at risk groups amongst breast cancer patients is essential for targeting survivorship care.

Our study showed that 34% of the participants experienced fatigue. Fatigue is a nonspecific symptom which affects about 70% of BCS [13]. It may affect women not only in the acute phase caused by the disease itself, but also by treatment and by psychological response to diagnosis [14]. It can also persist after treatment and stay throughout the patient’s life [13]. By recognizing this symptom early, interventions such as physical activity, counseling or addressing concomitant aggravators of fatigue can by initiated.

The second commonly reported symptom amongst the participating BCS is insomnia (33%). Evidence shows insomnia affects 30–75% of BCS and can cause significant distress in a patient’s life [15, 16]. Many studies have shown that patient’s baseline psychological and psychosocial status plays a key role in the rates of insomnia [15]. Some studies have also suggested that younger BCS have a higher tendency towards insomnia due to their tendency to be more concerned about diagnosis and treatment plan [15, 17]. Ensuring that questions about quality and quantity of sleep is are asked in crucial in follow up with BCS, as it can affect other common issues such as fatigue.

Hot flashes were the third most common side effect afflicting 26% of our patients. It can be explained by the gonadotoxic nature of the majority of breast cancer therapies [15, 18]. Again, females of post-menopausal age may experience this as a coincidence of natural menopause while younger patients will encounter hot flashes as a side effect of the therapy. In this younger population the psychosocial and physiological effects can be deleterious as the inhibition of ovarian function leads to sexual dysfunction, weight gain, sleep disturbances, atrophic vaginitis, night sweats, dyspareunia, and recurrent urinary tract infections [15, 19, 20]. Interestingly, hot flashes can disrupt sleep and decrease the percentage of deeper sleep stages [15]. Therefore, managing hot flashes either pharmacologically or psychosocially is essential.

Hot flashes were the third most common side effect afflicting 26% of our patients. It can be explained by the gonadotoxic nature of the majority of breast cancer therapies [15, 18]. Again, females of post-menopausal age may experience this as a coincidence of natural menopause while younger patients will encounter hot flashes as a side effect of the therapy. In this younger population the psychosocial and physiological effects can be deleterious as the inhibition of ovarian function leads to sexual dysfunction, weight gain, sleep disturbances, atrophic vaginitis, night sweats, dyspareunia, and recurrent urinary tract infections [15, 19, 20]. Interestingly, hot flashes can disrupt sleep and decrease the percentage of deeper sleep stages [15]. Therefore, managing hot flashes either pharmacologically or psychosocially is essential.

Other less commonly reported health issues amongst our breast cancer survivors were night sweats (23%), pain (22%), neuropathy (21%) and trouble concentrating (19%). Night sweats, pain, and neuropathy are likely related to cancer itself and anticancer treatment toxicity, while trouble concentrating is a part of psychological inadaptation to new diagnosis [21]. Though less common, these concerns are equally important to address as they can have serious implications of mental health and quality of life.

Based on our analysis, our program plans to initiate interventions directed towards younger breast cancer survivors and their specific concerns. We hope to provide additional education, workshops, and support groups that would address work/life balance, mental health, fertility, and sexual wellness. We also hope to provide support in a format that may be more conducive to their time constraints such as a virtual or social media format.

### Limitations and strengths

Our study represents a patient population from our institution that was surveyed during a specific time period and at a single institution. However, information was collected from a large number of participants with more than 900 people in a span of close to 5 years. In addition, patient characteristics of race and financial concerns were not included in this analysis. Additional social information such as smoking/alcohol history, functional status at baseline, and religious values were also not included. These additional socioeconomic factors and other considerations may also impact a patient’s QoL. The questionnaire was created to serve more as a discussion point for a clinical visit rather than used as a validated assessment tool. Lastly, more than half of the patients did not receive chemotherapy. This is reflective of the current treatment landscape from our national guidelines where fortunately most early staged breast cancer patients may omit chemotherapy if they have genomically low-risk hormone receptor-positive breast cancers. Our patients were evaluated only six months after their initial treatment. Our future plans for this survivorship program would include enrolling patients in a multi-modal program and subsequently evaluate for QoL changes.

## Conclusion

Our study results demonstrated concerns variably effecting breast cancer survivors. The results showed that younger survivors, patients with a higher stage at the time of diagnosis and receiving chemotherapy treatment seemed to be the patient population to significantly be affected by QOL issues. More advanced cancer stage and chemotherapy toxicity are known to cause more health-related and psychological distress in patients. In contrast, age-related differences in quality-of-life concerns are often underestimated in the complex care of BCS. In addition, common self-reported concerns reported included fatigue, insomnia and hot flashes. Identifying common concerns after treatment and at-risk populations are especially important to deliver quality care and to optimize interventions. We suggest that implementing specific recommendations gearing towards different age populations to existing guidelines for survivorship programs. More prospective studies are needed to address survivorship and to identify vulnerable populations among breast cancer survivors.

## Data Availability

The data that support the findings of this study are available from the corresponding author upon request.
